# Utilizing Cyclic Voltammetry to Understand the Energy Storage Mechanisms for Copper Oxide and its Graphene Oxide Hybrids as Lithium‐Ion Battery Anodes

**DOI:** 10.1002/cssc.201902784

**Published:** 2020-01-23

**Authors:** Cameron Day, Katie Greig, Alexander Massey, Jennifer Peake, David Crossley, Robert A. W. Dryfe

**Affiliations:** ^1^ William Blythe Ltd. Bridge Street, Church Accrington BB5 4PD United Kingdom; ^2^ National Graphene Institute University of Manchester Oxford Road Manchester M13 9PL United Kingdom; ^3^ Department of Chemistry University of Manchester Oxford Road Manchester M13 9PL United Kingdom; ^4^ Sir Henry Royce Institute for Advanced Materials University of Manchester Oxford Road Manchester M13 9PL United Kingdom

**Keywords:** anode materials, graphene, lithium-ion, metal oxide, cyclic voltammetry

## Abstract

Graphene‐based materials have been extensively researched as a means improve the electrochemical performance of transition metal oxides in Li‐ion battery applications, however an understanding of the effect of the different synthesis routes, and the factors underlying the oft‐stated better performance of the hybrid materials (compared to the pure metal oxides) is not always demonstrated. For the first time, we report a range of synthetic routes to produce graphene oxide (GO)‐coated CuO, micro‐particle/GO “bundles” as well as nano‐particulates decorated on GO sheets to enable a comparison with CuO and its carbon‐coated analogue, as confirmed using scanning electron microscopy (SEM) imaging and Raman spectroscopy. Cyclic voltammetry was utilized to probe the lithiation/delithiation mechanism of CuO by scanning at successively decreasing vertex potentials, uncovering the importance of a full reduction to Cu metal on the reduction step. The GO hybrid materials clearly show enhanced specific capacities and cycling stabilities comparative to the CuO, with the most promising material achieving a capacity of 746 mAh g^−1^ and capacity retention of 92 % after 30 cycles, which is the highest stable capacity quoted in literature for CuO. The simple cyclic voltammetry technique used in this work could be implemented to help further understand any conversion‐type anode materials, in turn accelerating the research and industrial development of conversion anodes.

## Graphical abstract

**Performance in preparation**: Graphene oxide/copper oxide composites are prepared using a variety of hydrothermal methods. The performance of these materials as lithium‐ion battery anodes is compared using charge–discharge and voltammetric measurements, to establish the barriers to reversible performance. The influence on preparation and electrode morphology on performance is explored.

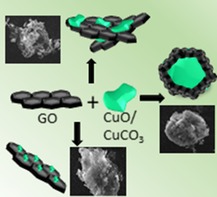

## Introduction

The performance demands of future energy storage applications have led to considerable research on alternatives to current electrode materials and battery chemistry. Although Li‐ion battery (LIB) capacity is limited by the cathode materials, significant effort is being expended to develop alternative anode materials to the industry standard, graphite. Graphite has been the anode material of choice for LIB since it was first utilized as a Li intercalation compound by Yazami and Touzain in 1983;^1^ however, this material suffers from safety issues when used in battery systems such as Li dendrite formation at the low lithiation potential of graphite (<0.1 V vs. Li/Li^+^),^2^ leading to potential thermal runaways. There are also environmental and supply concerns related to the mining of graphite, causing multiple closures to Chinese graphite mines in 2018.^3^

Transition metal oxides have been investigated extensively as an alternative LIB anode material to graphite, because of their high theoretical lithiation capacities, low cost and—in certain cases—ubiquity and low environmental “footprint”. CuO is a promising material in this regard as it fits the criteria above and has a theoretical capacity (674 mAh g^−1^) almost double that of graphite (372 mAh g^−1^).^4^ Unlike the insertion mechanism of graphite, transition metal oxides store Li ions via conversion reactions, which is depicted in its most general form below:

(1) $$\mathrm{MO}{}_{\left(s\right)}+2\;\mathrm{Li}{}^{+}{}_{\left(\mathrm{soln}\right)}+2\;e{}^{-}\vec{\leftarrow }M{}_{\left(s\right)}+\mathrm{Li}{}_{2}O{}_{\left(s\right)}$$

This multi‐electron transfer process is likely to proceed via two steps in the case of Cu, that is, reduction to Cu^I^, followed by formation of metallic Cu. The theoretical capacity of CuO has been calculated using Faraday's law assuming complete reduction to metallic Cu. A notable fade in specific capacity of CuO anodes is reported, which has been attributed to large expansion/contraction of particles during lithiation/delithiation cycles, leading to pulverisation of the electrode and a decrease in capacity.^5^

An important breakthrough in the understanding of transition metal oxides as LIB anodes was the seminal work from Tarascon's group, which demonstrated a strong correlation between increasing reversible capacity and metal oxide particle size, with the most stable performance seen when micron‐scale metal oxide particles were cycled, forming few nanometer diameter metal particles.^6^ Moreover, the voltage range of the cycled electrodes was found to strongly influence the cycling stability, with the best results obtained when the cells were cycled to the lowest (0.01 V vs. Li) voltage. A subsequent study by the same group used in situ transmission electron microscopy (TEM) to elucidate aspects of the reaction mechanism which, in the case of Cu, was suggested to consist of sequential reductions at 2.1, 1.6, and 1.2 V vs. Li, attributed to the formation of a partially lithiated phase (Li_*x*_CuO, *x*<0.4), CuO_2_ (when *x*>0.4), and Cu (when *x*>0.8), respectively. Extra capacity observed, that is, for *x*>2, was attributed to formation of an “organic film”.^7^ Another important contribution was made by Grey and co‐workers, who used synchrotron X‐ray and solid‐state NMR methods leading them to attribute the additional capacity to the reduction of the Li_2_O to LiH.^8^

Incorporation of graphene, or graphene oxide (GO), with CuO during electrode preparation has been found to increase the stable capacity and the cycling stability of the electrodes, although the reasons for this improvement in performance are not always obvious.^9^, ^10^ Clearly, introduction of a conducting support is beneficial, since CuO is a semiconducting material. Graphene will also help to stabilize smaller scale particles of CuO, which would otherwise be likely to aggregate and hence reduce the reversibility of the lithiation/delithiation process. An additional factor of potential relevance is the formation of the Li_2_O [see Eq. (1)], which possess very low electronic conductivity,^11^, ^12^ hence further inhibiting the reversibility of the lithiation process: again, small particles, corresponding to thinner Li_2_O layers, would mitigate this.

The specific changes in response to graphene incorporation are dependent on the respective morphologies of the metal oxide and graphene sheets, a zoo of differing geometries have been described.^13^ A relatively early report by Rai et al.^14^ described the formation of a reduced GO/CuO composite by a physical (milling) method. Although an initial capacity improvement was observed over the pure CuO, this decayed rapidly and within 20 cycles the capacity had fallen to the level of the pure metal oxide. A hydrothermal composite preparation also gave an improved capacity (ca. 500 mAh g^−1^, compared to ca. 300 mAh g^−1^ for the pure metal oxide), which showed a greater cycle stability.^15^ Clearly, given the foregoing discussion, a metal oxide with high surface area and intimate contact between metal oxide and graphene are necessary for an electrochemically reversible process. The effect of CuO microstructure on cell performance has been reported for the pure CuO (graphene‐free) case,^16^ but to the best of our knowledge, no systematic comparison between preparation method, electrode structure and cell performance has been reported for the composite case. In this work, we attempt to address this by presenting a systematic comparison of the performance of CuO/GO composite anodes prepared in three different ways, benchmarked against CuO and a carbon‐coated CuO. Particle morphology is compared with electrochemical performance, which includes half‐cell cycling data and cyclic voltammetry as a function of vertex potential, to assess the relationship between the extent of Cu reduction the reversibility of the reduction.

## Experimental Section

Cu–GO hybrid materials were prepared in five ways. Cu_2_(OH)_2_CO_3_ and GO were supplied by the industrial partner, William Blythe.

The sample denoted Cu‐1 consists of a CuO that was synthesized via a calcination process of Cu_2_(OH)_2_CO_3_. In short, Cu_2_(OH)_2_CO_3_ (4 g) was calcined in air at 300 °C for 4 h to produce CuO. Cu‐2 was produced by mixing CuO (3 g) from the Cu‐1 method and glucose (36 g) at room temperature for 4.5 h, filtering and then drying at 110 °C for 16 h. The dried product was then thermally treated in air at 300 °C for 4 h. Cu‐3 was produced by adding the commercially supplied Cu_2_(OH)_2_CO_3_ (5 g) to a GO dispersion (100 mL, 2.5 mg mL^−1^) and mixing for 3 h at room temperature. The product was then filtered, filter cake placed into the freezer at −16 °C for 16 h before freeze drying to prevent GO aggregation upon drying. The product was then calcined at 300 °C for 4 h.

Cu‐4 was synthesized by incorporating 5 wt % of GO into the synthesis of Cu_2_(OH)_2_CO_3_, filtering, then drying, the Cu_2_(OH)_2_CO_3_/GO hybrid in a vacuum oven at 110 °C overnight. The hybrid was then calcined in air at 300 °C for 4 h to produce CuO/GO.

Cu‐5 was produced by dissolving Cu(NO_3_)_2_.3H_2_O (3.2 g) and urea (1.21 g) in deionized water (50 mL). This solution was then slowly added to a GO dispersion (50 mL, 1.8 mg mL^−1^) with vigorous stirring and left to mix for 20 min. The solution was then transferred to a 200 mL Teflon‐lined stainless‐steel autoclave and treated at 130 °C for 12 h. The product was then filtered and washed until the conductance of the filtrate was below 100 μS, then placed into the freezer at −116 °C for 16 h. The frozen filter cake was then freeze dried to produce a fine powder of Cu_2_(OH)_2_CO_3_/GO. The composite was then calcined at 300 °C for 4 h to produce CuO/GO

The anode materials were characterized by scanning electron microscopy (SEM) performed on a FEI Quanta 650 FEG ESEM. X‐ray diffraction (XRD) was performed using a Proto AXRD Benchtop with 2*θ* scanned from 10° to 80° with Cu_Kα_ radiation. Raman spectra of the anode materials was recorded with a Renishaw inVia Raman Microscope, using 532 nm laser excitation and a 100× objective, operating at an intensity of 10 %. Thermal gravimetric analysis (TGA) was used to assess the relative carbon content and performed using a Mettler Toledo TGA/DSC 1 STAR^e^ System in the presence of air. Particle size was measured using a Malvern Mastersizer 2000 DLS particle sizer, where D50 is defined as the average particle diameter.

Electrochemical testing was completed in LIB half‐cells in a standard coin cell configuration versus a Li foil (all cell potentials quoted are therefore relative to this electrode). The anodes were produced by coating a slurry of Cu material, polyvinylidene difluoride (PVDF), and a commercial carbon black, Super‐P, (8:1:1) in *N*‐methyl pyrrolidinone onto a Cu current collector (14 μm thickness) before drying under vacuum at 80 °C for 16 h. Anode discs (15 mm) were punched and the 2023‐type coin cells were assembled in an Ar‐filled glovebox. The electrolyte was 1 m LiPF_6_ dissolved in a mixture of ethylene carbonate (EC) and dimethyl carbonate (DMC; 1:1 volume ratio). The mass loading and thickness of the anode were approximately 3 mg cm^−2^ and 60 μm, respectively. The PVDF was supplied by Kynar, Super‐P by Alfa Aesar and LiPF_6_ EC/DMC by Gelon LIB Group.

Cell performance was assessed via the capacity and cycling performance using a Basytec Cell Test System at a C‐rate of 0.2 C (based on the theoretical capacity of CuO), whereas cyclic voltammetry, recorded with an Autolab PGSTAT302N, was used to reveal some of the details of the reversibility of the anode reactions. Cyclic voltammograms are plotted as specific current (*I*_sp_), which is defined as the current per unit mass of electrode material versus cell voltage.

## Results and Discussion

**Figure 1:**
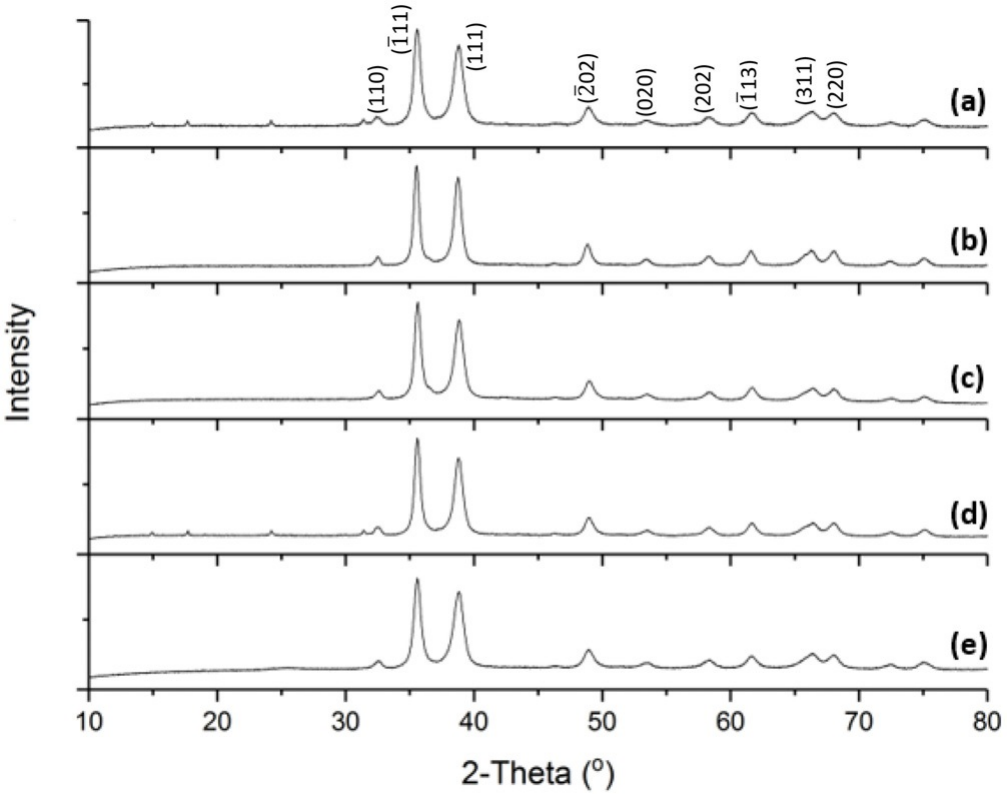
X‐ray diffraction patterns of a) Cu‐1, b) Cu‐2, c) Cu‐3, d) Cu‐4, and e) Cu‐5.

Figure 1 shows the XRD patterns of the CuO‐based electrode materials, which can all be indexed by the monoclinic symmetry (JCPDS Card No. 05 0661) described by the space group *C*2/*c*. The presence of small amounts (<5 % determined via extended calcination) of Cu_2_(OH)_2_CO_3_ is apparent in samples Cu‐1 and Cu‐4, based on the reflections seen at 2*θ*=15°, 18°, 24°, and 31°.^17^ Although undesirable, this material is expected to show similar chemistry to CuO, with respect to lithiation. We note that previous literature has described the use of transition metal carbonates an anode material.^18^ The absence of the GO peak at 2*θ*≈11° in Cu‐3, Cu‐4, and Cu‐5 indicates that the graphene is present as mono/few layers in the graphene hybrid materials.

**Figure 2:**
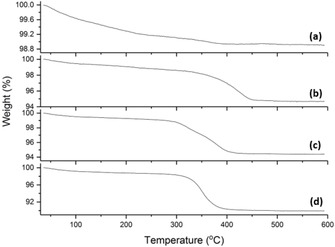
Thermogravimetric analysis of a) Cu‐2, b) Cu‐3, c) Cu‐4, and d) Cu‐5.

The carbon content of the Cu materials was confirmed with TGA as depicted in Figure 2. Cu‐2 had a very low carbon content, less than 1 wt %, based on the TGA data. In contrast, the carbon content of Cu‐3 was 5.2 %, Cu‐4 was 5.5 %, and Cu‐5 was 9.9 %. GO was introduced into Cu‐3 and Cu‐4 at 5 wt % during synthesis so this data confirms that all GO has successfully been incorporated into the material, enabling a direct comparison of the electrochemical performance of these materials. Cu‐5 has a graphene content close to 10 %, which, although it does not allow direct comparison to the other hybrids, is still an appropriate weight percentage when considering the expected specific capacity losses owing to reduced mass of active material, and also economic factors that come with introducing graphene into a battery material.

**Figure 3:**
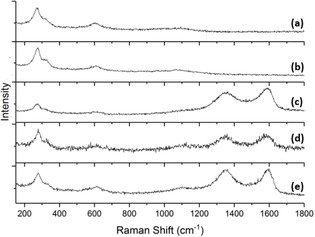
Raman spectra of a) Cu‐1, b) Cu‐2, c) Cu‐3, d) Cu‐4, and e) Cu‐5.

Raman spectroscopy was used to confirm the presence of graphene in the hybrid materials. Figure 3 a exhibits the three characteristic Raman active modes of CuO at 275 cm^−1^ (A_g_), 330 cm^−1^ (B_g_), and 610 cm^−1^ (B_g_),^19^ which are present in all Raman spectra. The three GO hybrid materials all display the characteristic D and G peaks of GO. The G peak at 1590 cm^−1^ corresponds to the in‐phase vibrations from the graphite lattice E_2g_ mode, whereas the D peak at 1350 cm^−1^ is caused by disorder on the basal plane created by the oxygen functional groups.^20^, ^21^

**Figure 4:**
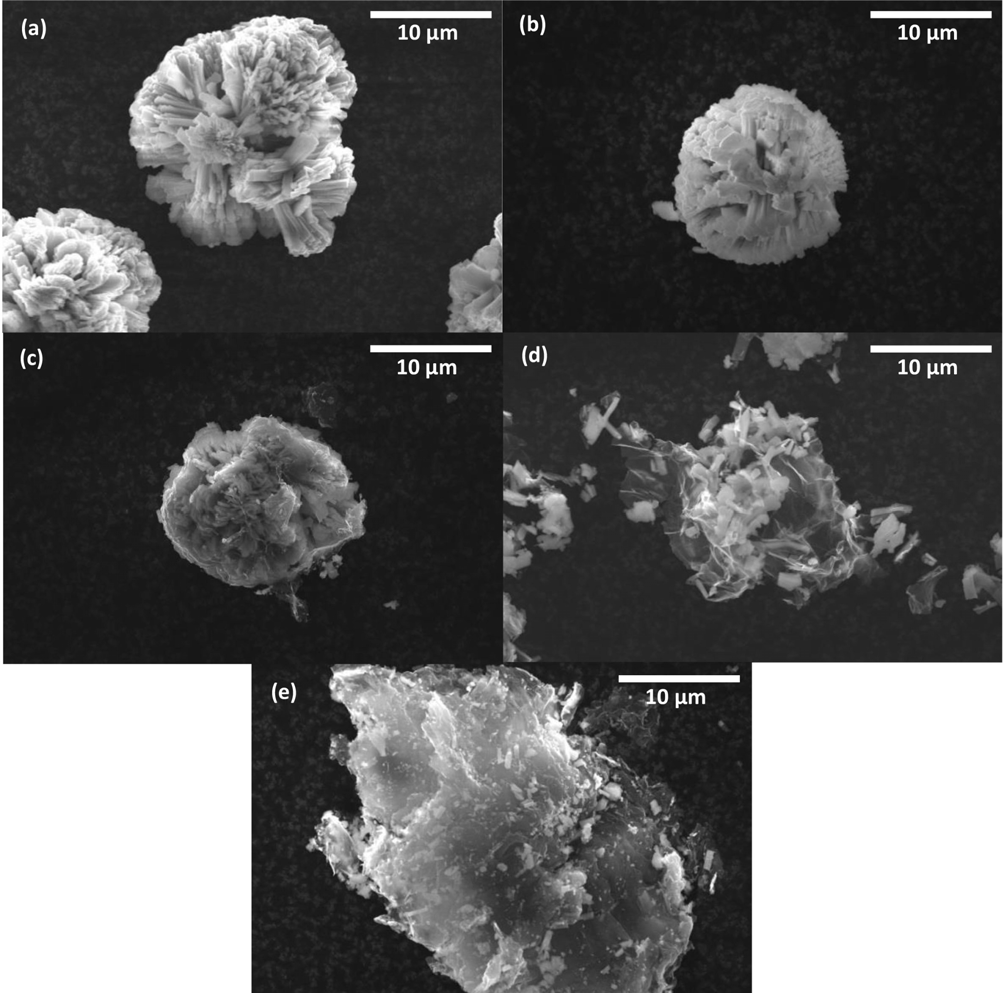
SEM images of the CuO/GO hybrids at 10 000× magnification a) Cu‐1, b) Cu‐2, c) Cu‐3, d) Cu‐4, and e) Cu‐5.

The morphology of the CuO‐based materials was compared using SEM imaging. Cu‐1 (Figure 4 a) depicts spherical secondary particles that consist of micro‐rods nucleating from a central point within the particle. The spherical nature of the CuO particles is desirable for battery applications as the spheres allow for better particle packing within the electrode, leading to greater connection between electrode components and therefore allowing greater volumetric energy densities to be achieved..^22^ The anisotropy of the microrods could also increase the rate capability of the electrode by providing shorter pathways for Li^+^ diffusion in and out of the particle. As expected, Cu‐2 (Figure 4 b) has a very similar morphology to Cu‐1 resulting from it being the same material with a low percent content of carbon coating. Carbon coating is a common technique used to increase the performance of battery materials by enhancing the electrical conductivity of the particles, so this material will enable the evaluation of the Cu‐3 graphene composite in comparison to industry standards.^23^

Cu‐3 (Figure 4 c) clearly shows wrapping of the particle in GO sheets, indicating that van der Waals forces are present between the carbon groups on the GO and the oxygen on the Cu allowing the hybrid particles to form during mixing. This encapsulation could have a 2‐fold benefit on the electrochemical performance of the materials. Firstly, by increasing the electronic conductivity of each particle enabling an increased charge transfer throughout the electrode, and secondly by providing a strong graphene coating that limits volumetric expansion. If realized, these effects would in turn enable greater energy densities and cycling stabilities to be achieved.^24^ Cu‐4 (Figure 4 d) has a vastly different morphology in comparison to Cu‐3, in which the synthesis procedure has inhibited the growth of these secondary primary particles to create hybrid structures of primary micro‐rod particles within GO sheets.

The segregation of the micro‐rods would further increase the 3 D conductive network of graphene within the electrode, potentially enabling even greater energy densities to be achieved.^25^ Hybrid Cu‐5 was synthesized via hydrothermally precipitating nanoparticles of CuCO_3_ onto GO sheets as can be seen in Figure 4 e. The production of nanoparticles could increase the reaction kinetics for the decomposition of the usually unreactive Li_2_O on the re‐oxidation of Cu.^26^ However, as previously stated, a strong correlation between CuO particle size and cycling stability was reported in the work of Grugeon et al., with the nanometer‐scale oxide particles having a detrimental effect on cycling performance.^27^

**Figure 5:**
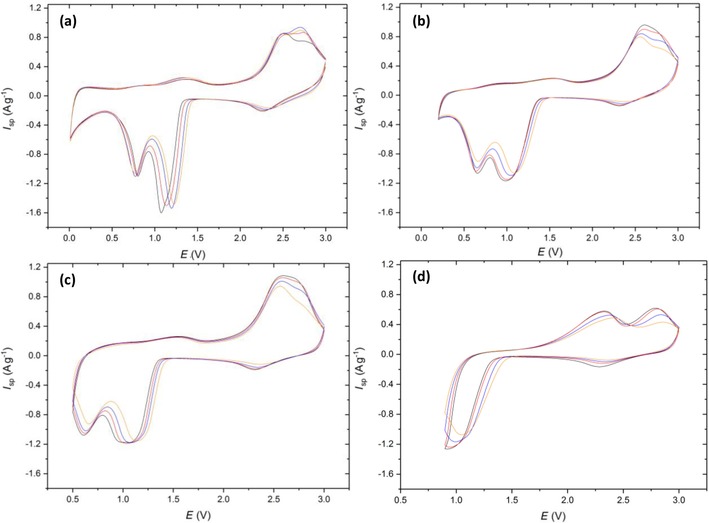
Cyclic voltammetry of Cu‐1 scanned at 0.5 mV s^−1^ from 3 V to vertex potentials of a) 0.01, b) 0.2, c) 0.5, and d) 0.9 V. Colors correspond to cycle number 1 (black), 2 (red), 5 (blue), and 10 (orange).

As noted in the introduction, the mechanism of CuO reduction in the LIB cell has been discussed by Debart et al.,^7^ using in situ TEM to aid their interpretation of the electrochemical data. Figure 5 presents the cyclic voltammetric data for the CuO electrode. The small “pre‐peak” seen at approximately 2.3 V corresponds to a similar feature observed at a similar potential in Ref. 7. The retention of CuO structure led to the assignment of a limited lithiation of the oxide. The second reduction feature, a much more prominent one with a peak potential in the range 1.2 to 1.0 V, was attributed to Cu_2_O by Debart et al. By contrast, the work of Wang et al.,^28^ who used in situ TEM to study lithiation of CuO nanowires, suggested that the first cycle lithiation involved a conversion from CuO to Cu, which on delithiation, disproportionates to Cu_2_O and CuO.

**Figure 6:**
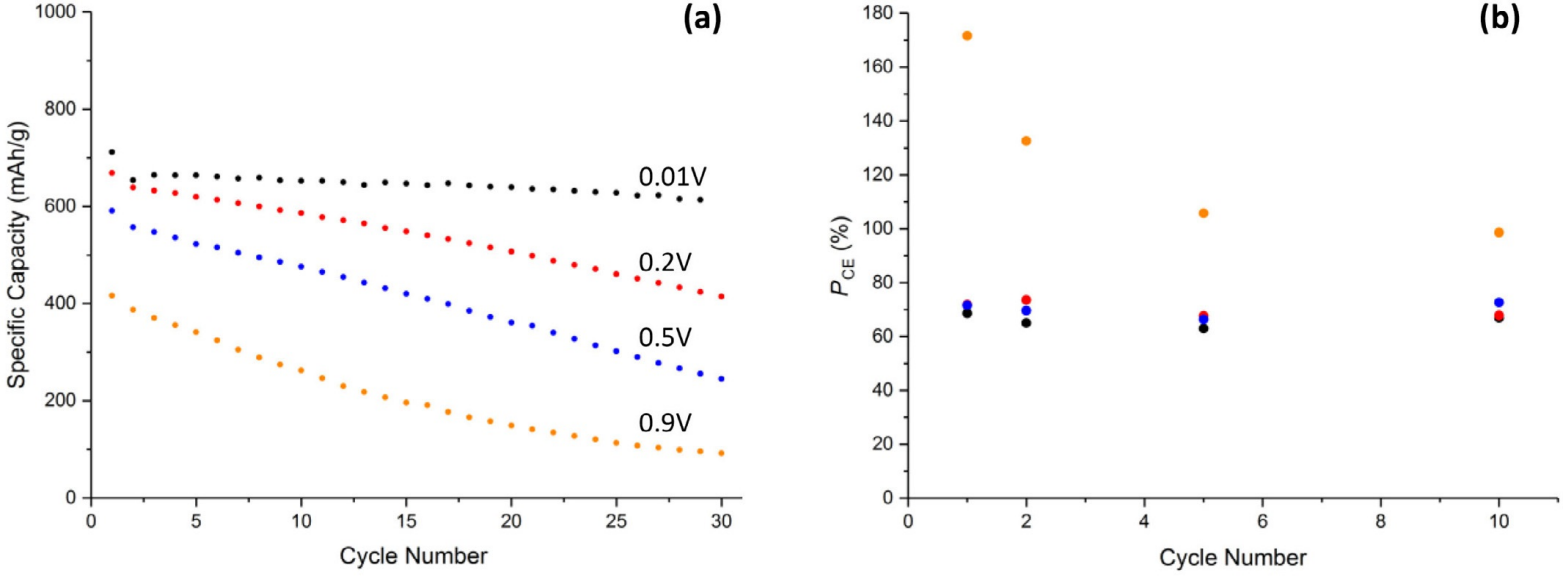
a) Galvanostatic cycling data of Cu‐1 at 0.2 C and b) potentiostatic coulombic efficiency at the various lower vertex potentials. Potentials stated in (a) correspond to the same color points in (b).

Note that, for all the voltage ranges explored in Figure 5, a prominent difference between the first and subsequent voltammetric cycles is not observed, which supports the mechanism proposed by Tarascon and co‐workers,^7^ rather than disproportionation, on the lithiation cycle. The charge associated with the discharge cycle is approximately independent of cycle number at all vertex potentials; however, the reduction peaks are “sharpened” (i.e., occur over a narrow potential range) by proceeding to lower vertex potentials. This sharpening could be a result of a greater amount of residual Cu and Cu_2_O present after the oxidative sweep arising from an increase in the amount of CuO reduction at lower potentials (as evidenced by the increased specific capacities in Figure 6 a). The presence of Cu and Cu_2_O crystallographic phases will increase the rate of reduction of the CuO, and the Cu will increase the conductivity of the electrode, leading to the reactions occurring within a more limited potential and sharper voltammetric peaks.

In a separate publication, Laik et al. calculated the cell potentials (i.e., thermodynamics of reduction of metal oxides to metals, relative to Li oxidation), showing that the reduction of a range of transition metal oxides is thermodynamically feasible, a reduction potential of 2.22 V was calculated for the case of CuO vs. Li^+^/Li. These authors note that all of these potentials are considerably higher than the observed voltages (c.f. 1.0–1.2 V above for this process), a discrepancy attributed to the slow kinetics of the conversion reaction.^29^

The second prominent reduction peak at 0.6 V, only visible in Figure 5 a–c, is attributed to Cu_2_O reduction to Cu. One peculiarity of the LIB system is that the only “sink” for the oxide ions, formally generated on the reduction of Cu, is the incoming Li^+^ ion, as protons are absent and the battery solvent only decomposes at potentials below those of cuprous/cupric ion reduction. Finally, a small feature is observed at potentials below 0.4 V: this is consistent with the additional charge attributed by Tarascon and co‐workers^7^ to the formation of an “organic film”, but later reported by Grey and co‐workers to be linked with further reduction of the lithiated products to LiH as a new “inorganic” layer.^8^ The reverse part of the cyclic voltammograms of Figure 5, corresponding to the delithiation process, shows that the main delithiation process takes place at cell voltages above 2.0 V. A small pre‐peak is seen for all vertices lower than or equal to 0.9 V, that is, for all cases except Figure 5 d. Tarascon and co‐workers^7^ attributed this feature to the reversal of the “organic film” formation process seen at the lowest voltages, but the observation of this pre‐peak on the reverse cycle at a vertex potential of 0.5 V (Figure 5 c) suggests that it is not directly related to the “extra” charge formation process seen at <0.4 V. The position of this peak is, however, vertex dependent: its maximum lies between 1.3 and 1.6 V, with the shift to the more positive voltages seen for the increased (more positive) vertex potentials. This indicates that this oxidative pre‐peak could be a result of the reversible formation and decomposition of an solid electrolyte interface (SEI) layer, which typically forms below 0.8 V.^30^ Changing the vertex potential to 0.9 V supports this argument as it is only in this cyclic voltammogram that the oxidative pre‐peak is absent. The “organic” layer described by Tarascon is therefore more likely to be typical SEI formation, which begins to form at 0.8 V, and the extra capacity at <0.2 V the inorganic layer as described by Grey and co‐workers.^8^

The other notable feature of the “reverse” part of the CVs is the splitting of the main delithiation process into two separate voltammetric peaks, which is seen most prominently for the highest vertex (Figure 5 d). The observation of a “merged” delithiation process is at variance with the reference 7 and suggests that the larger particles used here undergo a single‐step re‐oxidation of metallic Cu to CuO. Interestingly, the more pronounced “split” peak seen in Figure 5 d suggests that the Cu_2_O species undergoes disproportionation to generate Cu and CuO, with the co‐existence of these species favoring a two‐step delithiation.

A calculation of the charge/discharge ratio from the potentiostatic data at the different cycle numbers is shown in Figure 6 b. At the lower vertex potentials (0.01, 0.2, and 0.5 V) the coulombic efficiencies are in the range of 60–70 %, suggesting an incomplete conversion of Cu back to CuO. This suggests that the degradation pathway for CuO anodes proceeds via incomplete oxidation of Cu and coupled with—possibly driven by—incomplete conversion of Li_2_O back to Li^+^, resulting in excess insulating Li_2_O remaining at the anode. This removes Li from the system and decreases the electrical conductivity of the electrode.

However, at 0.9 V the coulombic efficiency is as high as 171 % on the 1^st^ cycle and 132 % on the 2^nd^ before decreasing to 97 % on the 10^th^ cycle. This extra charge gain was only measured in the potentiostatic mode; galvanostatically the coulombic efficiency remained below 100 % on all cycles. Coulombic efficiencies greater than 100 % can typically be attributed to undesired side reactions/shuttling processes, the latter being common in other cell systems such as a LiS.^31^ Although it is unlikely that shuttling would occur in this system, a dissolution of CuO and plating of Cu^2+^ to Cu on the electrode prior to charging would give rise to a larger portion of reduced Cu available for oxidation to give the two peaks as depicted in Figure 5 d. This theory is however speculative and would require further investigation to understand the mechanism giving rise to this additional capacity.

Figure 6 a shows the specific capacity at each potential range as a function of cycle number. At 0.01 V an initial capacity of 711 mAh g^−1^ was obtained being greater than the theoretical (674 mAh g^−1^), presumably as a result of the inorganic layer formation. This then drops to 654 mAh g^−1^ with a capacity retention of 86 % after 30 cycles. As expected, increasing the lower vertex potential decreases the specific capacity as the extent of lithiation that occurs in that potential window is decreased. When the vertex potential was increased to 0.2 V a similar initial capacity was obtained; however, the capacity on subsequent cycles decreased at a much quicker rate (62 % retention) in comparison to the 0.01 V cell. Considering that the only known mechanism occurring below 0.2 V is the formation of the additional inorganic layer, these observations suggest that this new inorganic layer is crucial to the cycling stability. This capacity retention degrades at even faster rates at the 0.5 V (41 % retention) and 0.9 V (22 % retention) vertex potentials. An explanation for the low retention at 0.9 V could be that if the disproportionation of Cu_2_O to Cu and CuO does occur, then this decreases the fraction of Cu^II^ available for reduction on subsequent cycles, introducing a new degradation pathway for the electrode. Alternative reasoning could be owing to the lack of SEI layer formation, which is known to enhance the cyclability of an anode material if a stable SEI is formed.^32^

**Figure 7:**
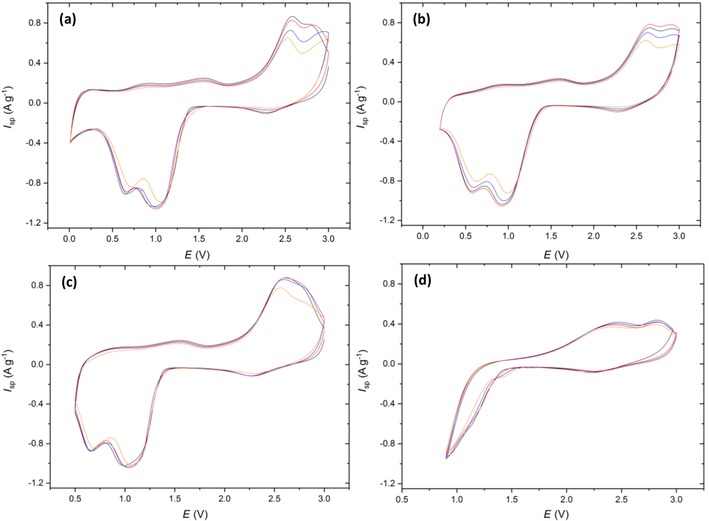
Cyclic voltammetry of Cu‐2 scanned at 0.5 mV s^−1^ from 3 V to vertex potentials of a) 0.01, b) 0.2, c) 0.5, and d) 0.9 V. Colors correspond to cycle number 1 (black), 2 (red), 5 (blue), and 10 (orange).

Cu‐2 was investigated through the same cyclic voltammetry testing procedure (Figure 7): the discussion of the mechanism is related to the conclusions drawn for Cu‐1. Distinct differences are present at the 0.01 and 0.2 V vertex potentials when compared to Cu‐1. Firstly, the reduction peaks at 1 and 0.6 V are much broader and overlap to almost form one reduction peak. Secondly, the oxidation peaks that typically arise ≈2.5 V have become more separated with the Cu_2_O to CuO conversion becoming less favorable as highlighted by the shift in peak maxima to higher potentials. It appears that the carbon coating has reduced the reversibility of the conversion reactions at the lower vertex potentials, with the electronic conductivity enhancements being outweighed by the extra impedance to Li^+^ ion diffusion out of the particles after the initial Cu oxidation.

**Figure 8:**
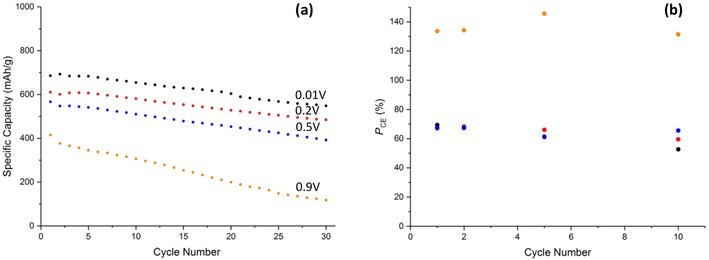
a) Galvanostatic cycling data of Cu‐2 at 0.2 C and b) potentiostatic coulombic efficiency at the various lower vertex potentials. Potentials stated in (a) correspond to the same color points in (b).

A common feature at the 0.9 V (Figure 7 d) vertex is the presence of the two oxidative peaks at 2.4 and 2.8 V, further supporting the hypothesis of Cu_2_O disproportionation. These peaks again give rise to a potentiostatic coulombic efficiency (Figure 8 b) greater than 100 % on all cycles, demonstrating that this feature is not an artefact of the Cu‐1 material, electrode or cell, but common throughout multiple materials at the 0.9 V vertex.

An investigation into the cyclability of Cu‐2 yielded poorer results than for the uncoated material at the 0.01 V vertex with an initial capacity of 686 mAh g^−1^ and a retention of 80 % after 30 cycles. This confirms the findings from the CVs that indicated a poorer reversibility at the lowest vertex potential. The presence of the carbon coating did however increase the cyclability of the CuO in the remaining 0.2 (80 %) 0.5 (70 %), and 0.9 V (28 %) when compared to Cu‐1, concurring with what is commonly quoted in literature.^24^ It could be that there are unfavorable interactions between the inorganic layer that forms below 0.2 V and the carbon coating that causes this degradation.

**Figure 9:**
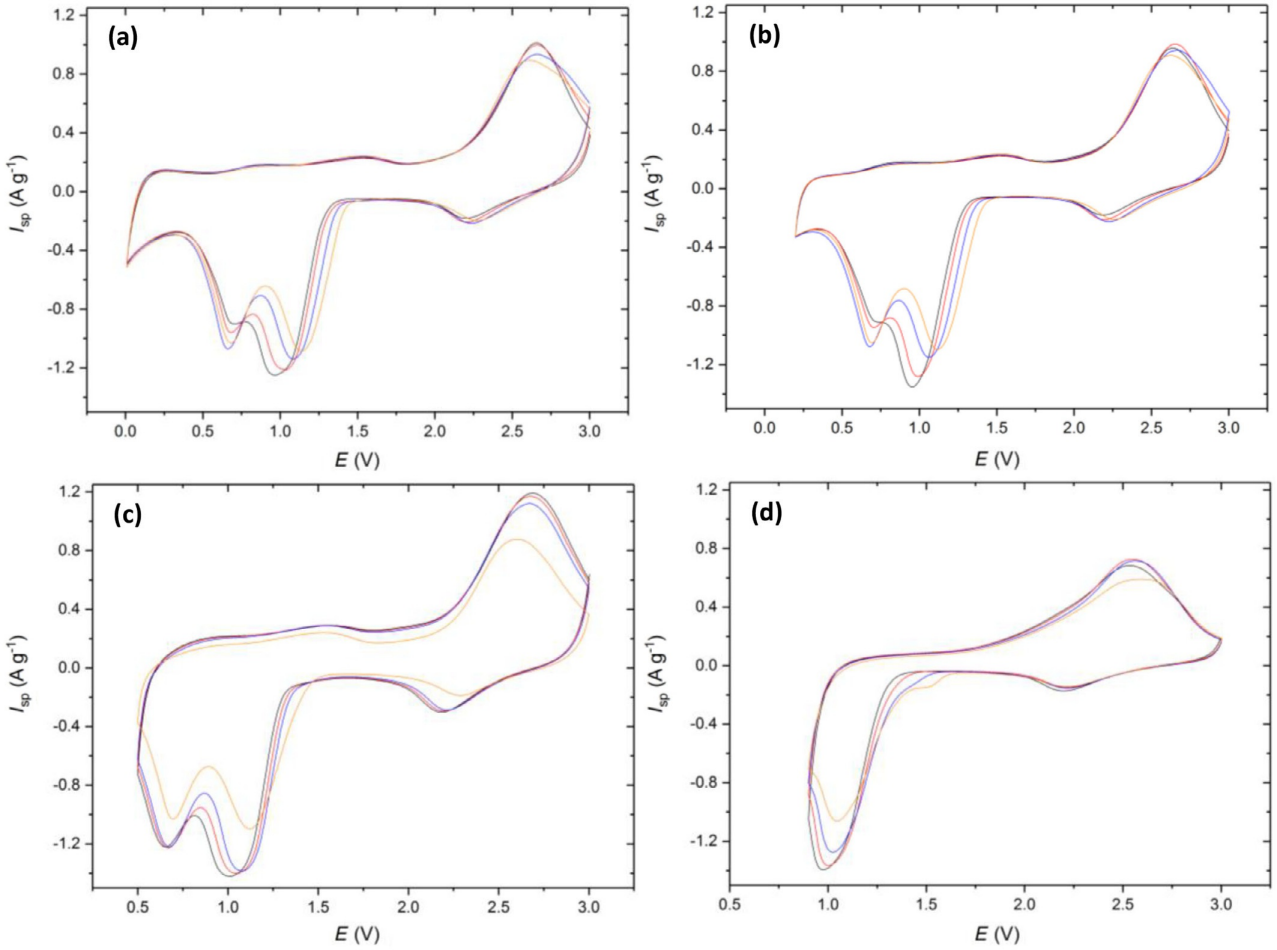
Cyclic voltammetry of Cu‐3 scanned at 0.5 mV s^−1^ from 3 V to vertex potentials of a) 0.01, b) 0.2, c) 0.5, and d) 0.9 V. Colors correspond to cycle number 1 (black), 2 (red), 5 (blue) and 10 (orange).

Figure 9 exhibits the cyclic voltammograms of Cu‐3, the GO‐coated CuO composite. At a glance, the lowest vertex potential of 0.01 V (Figure 9 a) displays a similar shape to Cu‐1, with reduction peaks at 2.3, 1.0, and 0.6 V, the latter two shift to higher potentials with increasing cycle number, therefore becoming more disfavored. This indicates that the GO coating does not give rise to a 100 % capacity retention. One potential improvement to the cycling stability when compared to Cu‐1 is the that the peak at 0.6 V corresponding to the reduction of Cu_2_O to Cu does not shift to a lower potential as the vertex potential increases from 0.01 to 0.9 V. Another feature to note in Figure 9 a is the merging of the oxidation peaks instantaneously at 2.6 V, suggesting that the GO coating is enhancing the single‐step oxidation of Cu to CuO.

It was previously reported that graphene coatings can enhance the electron charge transfer and Li^+^ diffusivity in and out of active materials;^33^ therefore, the GO could be playing both roles in this system, enabling greater accessibility to unreacted Li_2_O and an increase in reaction rate. This peak merging is concurrent at all vertex potentials, in contrast to what is seen at 0.9 V in the Cu‐1 material. This suggests that the only process occurring in Figure 9 d is the re‐oxidation of the Cu_2_O back to CuO at 2.6 V without the disproportionation of the Cu_2_O to CuO and Cu that was proposed in Cu‐1.

**Figure 10:**
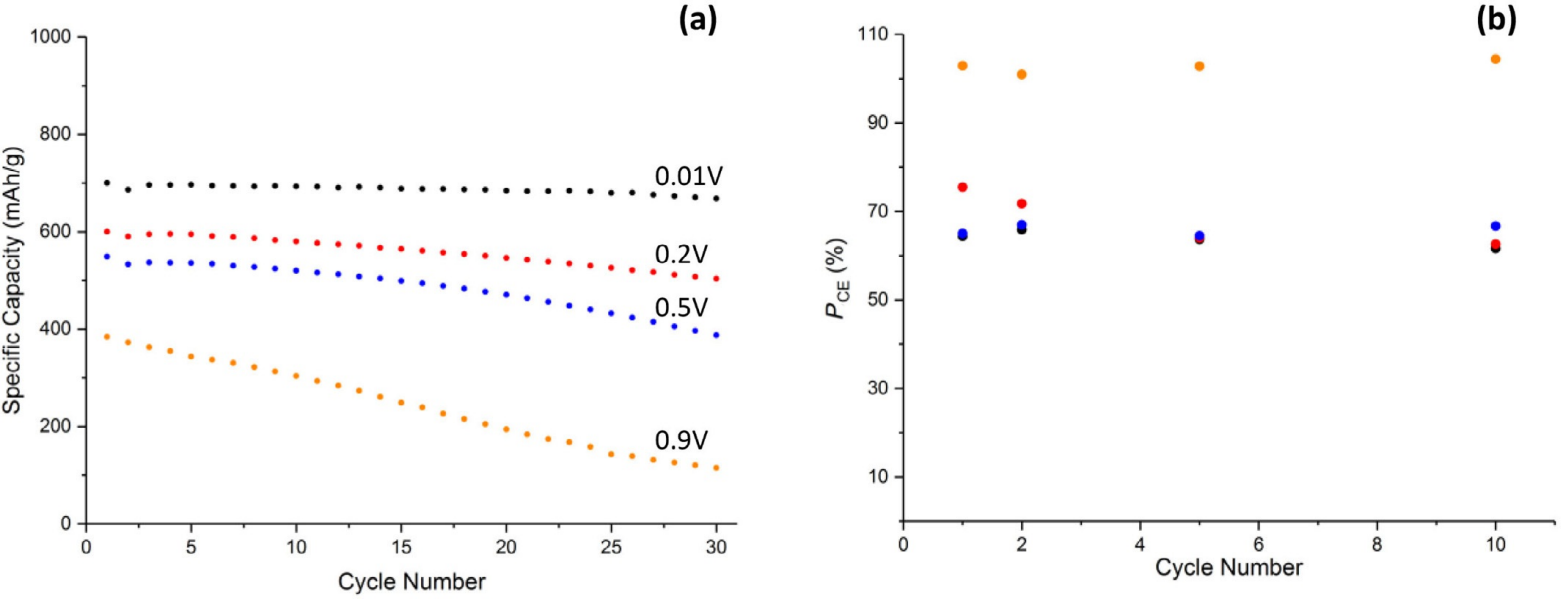
a) Galvanostatic cycling data of Cu‐3 at 0.2 C and b) potentiostatic coulombic efficiency at the various lower vertex potentials. Potentials stated in (a) correspond to the same color points in (b).

The potentiostatic coulombic efficiency (Figure 10 c) remained between 100–104 % on all cycles. When taking into account the error in determining the area under the curve for separate charge and discharge steps, this result is not significant enough to conclude that extra capacity is being gained on the charge step. This suggests a strong correlation between the two‐stage oxidation in Figure 5 d and the extra capacity gained on the charge step giving rise to a>100 % coulombic efficiency. Another discrepancy for the 0.9 V vertex in Figure 9 d, compared to Figure 5 d, is the emergence of a reduction “pre‐peak” at 1.5 V on the 10^th^ cycle. This peak could be attributed to stronger interactions that form between the GO and CuO during cycling. Previous studies have shown that greater oxidative catalytic activities can be achieved for CuO when combined with GO to form a hybrid material.^34^, ^35^ Therefore, the Cu atoms on the surface of the CuO particle in contact with GO could be undergoing a faster rate of reduction to Cu_2_O hence the process is more favorable at the higher potential. One final point to note is the SEI decomposition that arises on the oxidative scan. With Cu‐3 there are two clearly defined oxidative peaks at 0.9 and 1.5 V (note that these are also seen with Cu‐1 but are less defined) suggesting that there are two different chemical compositions of SEI forming on the CuO, which in turn decompose at different potentials.

Figure 10 a presents the cyclability of the Cu‐3 hybrid material. At the lowest vertex potential of 0.01 V an initial capacity of 700 mAh g^−1^ was obtained with a 95 % retention of this capacity after 30 cycles, indicating that the GO coating has had a significant benefit to the cycling stability of the CuO. The slightly lower initial capacity here compared with Cu‐1 is most likely attributed to the lower mass of active CuO material (95 % of the mass of the hybrid). This increased cycling stability is also realized when cycled to the 0.2 and 0.5 V vertex potentials (84 % and 70 % retention, respectively), supporting the above CV data that suggests that the GO enhances the reversibility of CuO reduction/oxidation at higher vertex potentials.

An explanation for the loss in capacity for the first three vertex potentials can be determined by considering the potentiostatic coulombic efficiency (Figure 10 b), which again show variances between 60–75 % for all cycles suggesting an incomplete conversion of Cu back to CuO, leaving unreacted Li_2_O at the electrode. At 0.9 V the capacity retention after 30 cycles is 30 %, which is only 8 % higher than for the pure Cu‐1. This indicates that accelerated degradation could arise from a lack of SEI formation, the CuO not being fully reduced to the Cu metal or via another unknown process.

**Figure 11:**
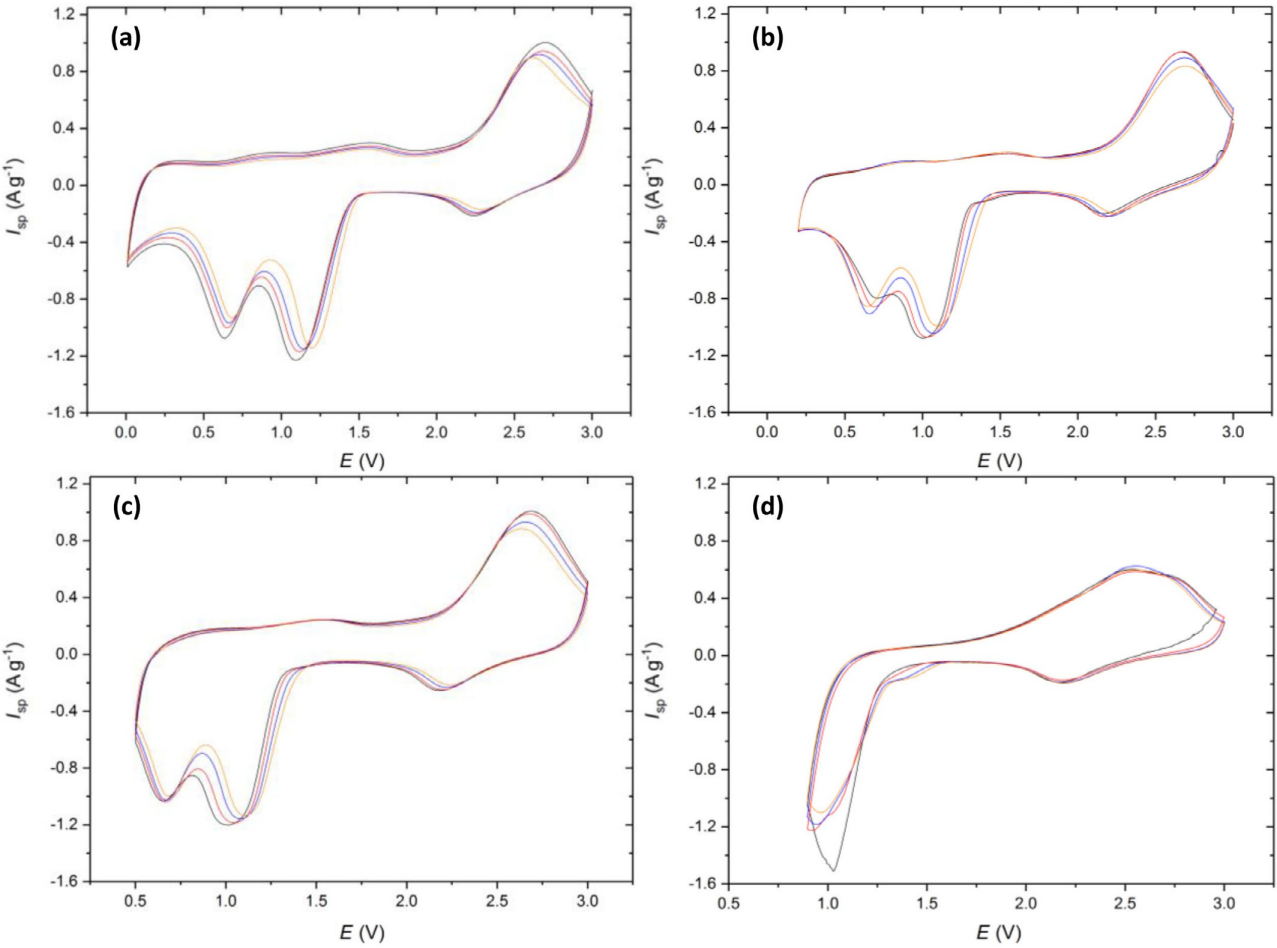
Cyclic voltammetry of Cu‐4 scanned at 0.5 mV s^−1^ from 3 V to vertex potentials of a) 0.01, b) 0.2, c) 0.5, and d) 0.9 V. Colors correspond to cycle number 1 (black), 2 (red), 5 (blue) and 10 (orange).

Figure 11 displays the CV potential ranges for the Cu‐4 hybrid that displayed a primary micron‐sized CuO within GO “bundles” morphology. The CVs in Figure 11 a–c show very similar shapes to that in Figure 9 a–c, indicating that similar CuO/GO interactions are occurring, causing the reduction peak at 0.7 V to remain at the same potential when the vertex potential increases. In contrast to Cu‐3 at the 0.9 V vertex (Figure 11 d), the oxidative peak at 2.6 V is much broader and could also be considered to consist of two distinct oxidative peaks with similar maxim oxidative peaks with similar maximum potentials. One other notable difference for Cu‐4 is seen at the 0.9 V vertex potential in Figure 11 d. The additional reductive peak at 1.4 V is now visible after the earlier 5^th^ cycle compared to the 10^th^ in Figure 9 d. This can be rationalized after inspection of the morphology of Cu‐4 in Figure 4 d, which displays a more intimate mix of the primary micro‐rods particles within a GO “bundle”, which could speed up the rate at which this accelerated reduction of CuO to Cu_2_O occurs.

**Figure 12:**
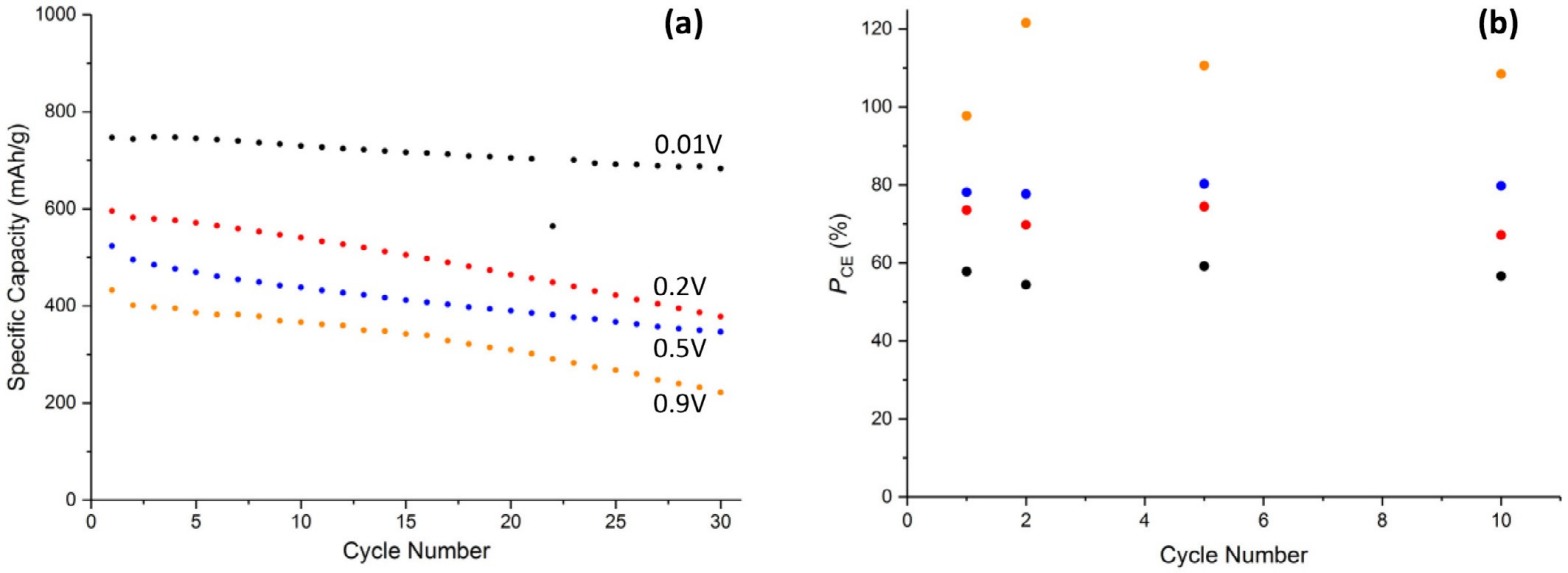
a) Galvanostatic cycling data of Cu‐4 at 0.2 C and b) potentiostatic coulombic efficiency at the various lower vertex potentials. Potentials stated in (a) correspond to the same color points in (b).

The cycling stability of Cu‐4 is displayed in Figure 12 a. At the lowest 0.01 V vertex potential, a specific capacity of 746 mAh g^−1^ was measured with a 92 % capacity retention after 30 cycles. A number of articles quote values >1000 mAh g^−1^ for the first cycle for CuO graphene hybrids; however, this then drops below 600 mAh g^−1^ after the 1^st^ cycle. Therefore, to the best of our knowledge we believe this to be the only CuO/graphene material with a stable additional capacity.

The cause of this additional capacity could be 2‐fold: (i) the reduction of LiOH by Li^+^ on CuO surface to form LiH and Li_2_O and (ii) the intercalation of Li^+^ between the GO sheets as demonstrated by Manthiram and co‐workers,^36^ which allows for capacities of over 800 mAh g^−1^ to be obtained as a result of increased interlayer spacing. This Li^+^ intercalation however should be visible on the CV as a clearly defined oxidative peak at 0.25 V; this is not observed in Figure 11 a. The extra capacity must be attributed to the inorganic layer formation occurring below 0.2 V. A justification as to why this higher capacity was obtained for Cu‐4 and not Cu‐3 could be particle size related. Cu‐4 has a much lower d50 (median value of the particle size distribution; here: 8.5 μm) compared to Cu‐3 (26 μm), which could lead to a great accessibility to the surface of the particles, allowing for a larger inorganic layer to be formed and therefore greater lithium storage.

For the increased vertex potentials of 0.2, 0.5, and 0.9 V, capacity retentions of 63 %, 66 % and 51 % were obtained, respectively. The cycling stability at vertex 0.9 V is much higher than all other materials and the high reversibility of the CV in Figure 11 d is also in agreement with this increased galvanostatic cycling stability. A reason for this could be related to the GO acting as an artificial SEI as a result of the primary particle coverage as seen in Figure 4 d. It is common knowledge that the formation of a stable SEI layer can enhance the cyclability of anode materials such as graphite and silicon;^32^ however, at the 0.9 V vertex we expect no SEI formation. Chen and co‐workers demonstrated the use of GO‐based materials to form artificial SEI layers on transition metal oxides for use in LIBs,^37^ so this type of artificial SEI could also have formed in Cu‐4. We would not see this in the previous composite materials as the carbon/graphene coatings were only present on the surface of the secondary particles and therefore would be an incomplete coverage of all the available particle surface for SEI formation. It can also be noted that the potentiostatic coulombic efficiency (Figure 12 b) at the 0.9 V vertex remains above 100 % after the first cycle, further supporting the hypothesis of enhanced stability.

**Figure 13:**
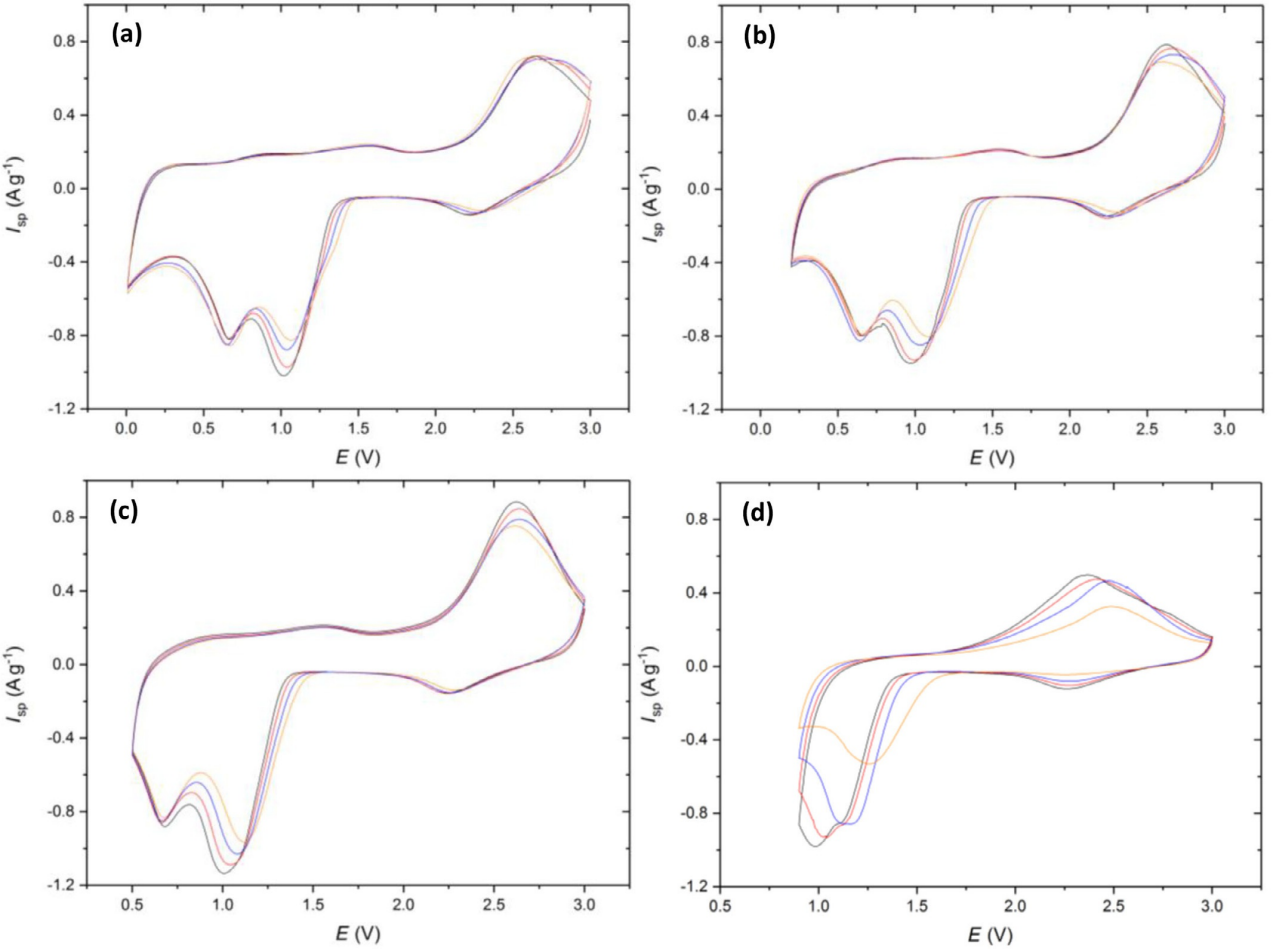
Cyclic voltammetry of Cu‐5 scanned at 0.5 mV s^−1^ from 3 V to vertex potentials of a) 0.01, b) 0.2, c) 0.5, and d) 0.9 V. Colors correspond to cycle number 1 (black), 2 (red), 5 (blue) and 10 (orange).

Figure 13 displays the cyclic voltammetry for final hybrid material: Cu‐5, which possessed the morphology of nano‐sized CuO particles deposited on the surface of GO sheets. Generally, the CVs show a similar shape to the other graphene hybrids in having little dependence of peak position on vertex potential, and in the merging of the oxidative peak at 2.35 V. The cyclic voltammograms do appear to be more reversible than all materials in Figure 13 a–c,; however, this stability collapses at the 0.9 V vertex. The peak maximum of the merged oxidative peak (Figure 13 d) is much lower than in the other CuO materials, suggesting the oxidation of Cu_2_O back to CuO is more favorable in Cu‐5 in the first cycle. However, in subsequent cycles the delithiation peak shifts back to 2.5 V as the overall storage capacity decreases. It can also be noted that at this 0.9 V vertex we do not see the additional reductive peak at 1.5 V that was observed in Cu‐3 and Cu‐4, indicating a less intimate mix of the CuO and GO. The coulombic efficiency at this vertex again exceeds 100 %, reaching 109 % in the 1^st^ cycle before decreasing steadily to 84 % by the 10^th^ cycle in agreement with the other four materials. This instability at 0.9 V could be attributed to an incomplete coverage of CuO nanoparticles, which seem to have deposited onto the GO sheets rather than be embedded within a GO structure as can be confirmed for Cu‐4.

**Figure 14:**
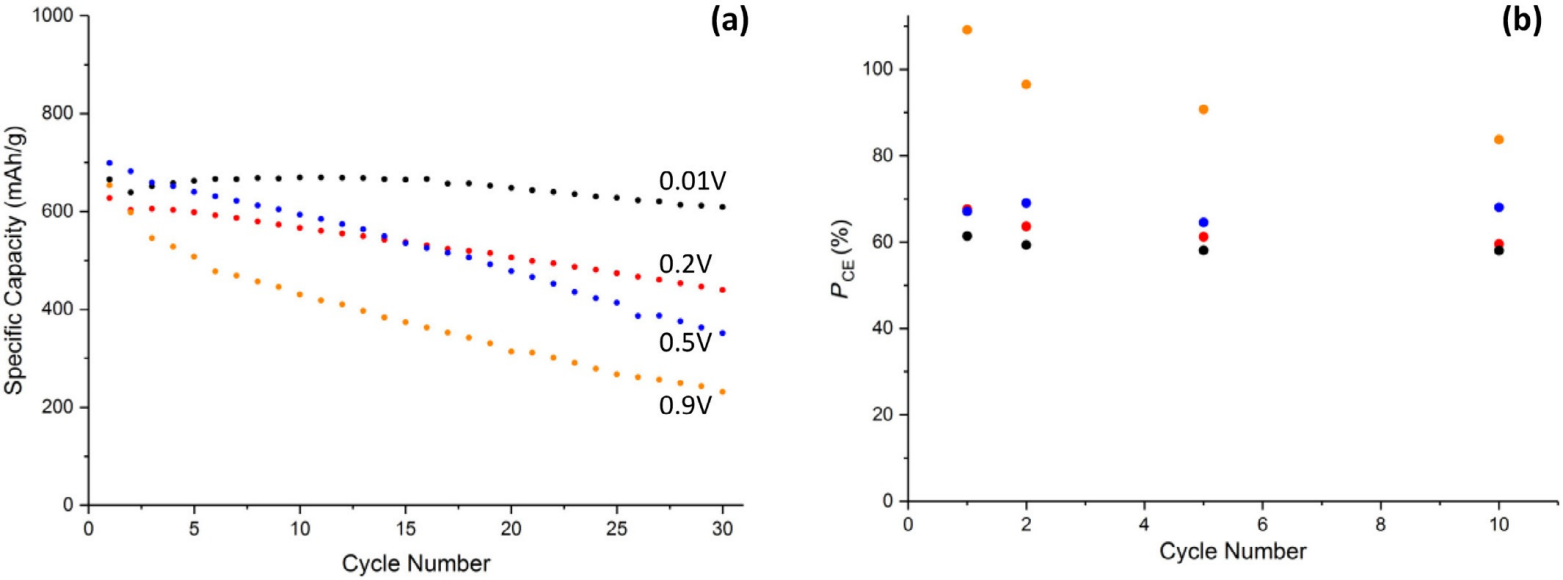
a) Galvanostatic cycling data of Cu‐5 at 0.2 C and b) potentiostatic coulombic efficiency at the various lower vertex potentials. Potentials stated in (a) correspond to the same color points in (b).

The initial capacity of the 0.01 V vertex potential was 665 mAh g^−1^ and retained 91 % of this capacity after 30 cycles (Figure 14). This is the lowest capacity achieved of all materials presented in this work; however, these cannot be directly compared because of the higher content of GO present in Cu‐5, leading to an overall decrease in active material mass. Despite having a similar morphology to Cu‐4, the cycling stability of Cu‐5 is poorer at most vertex potentials (70 % at 0.2 V, 50 % at 0.5 V, and 35 % at 0.9 V), which is in agreement with the Tarascon article^27^ demonstrating a poorer cyclability with decreased particle size.

## Conclusions

We describe a systematic comparison of CuO/graphene oxide (GO) composite materials for use as anodes in Li‐ion batteries synthesized via coating, precipitation, and hydrothermal routes to produce a variety of morphologies, alongside a comparison with a pure CuO and a carbon‐coated CuO as is the current industry standard. Experimentally we have realized clear performance enhancements in both specific capacity and cycle stability through the incorporation of GO in all three routes. Whereas a GO coating increased the cyclability of the CuO/GO hybrid, it was the precipitation route that produced the highest stable specific capacity know in literature at 746 mAh g^−1^ at 0.2 C. Using cyclic voltammetry at four different voltage ranges we have also further probed the lithiation/delithiation mechanism of CuO, which has in turn enhanced understanding of why the graphene hybrids possess greater electrochemical performances in Li‐ion battery applications. The advanced CuO/GO material offers a potential environmentally benign alternative to the graphite anode as well as a method to investigate the mechanism of transition metal oxides using a simple cyclic voltammetry technique.

## Conflict of interest

*The authors declare no conflict of interest*.
